# Detoxification of the Fumonisin Mycotoxins in Maize: An Enzymatic Approach

**DOI:** 10.3390/toxins11090523

**Published:** 2019-09-10

**Authors:** Johanna Alberts, Gerd Schatzmayr, Wulf-Dieter Moll, Ibtisaam Davids, John Rheeder, Hester-Mari Burger, Gordon Shephard, Wentzel Gelderblom

**Affiliations:** 1 Mycotoxicology Research Group, Institute of Biomedical and Microbial Biotechnology, Cape Peninsula University of Technology, Bellville 7535, South Africa; 2 BIOMIN Research Center, BIOMIN, Technopark 1, 3430 Tulln, Austria; 3 Department of Biomedical Science, University of the Western Cape, Bellville 7535, South Africa; 4 Department of Biochemistry, Stellenbosch University, Stellenbosch 7600, South Africa

**Keywords:** fumonisin, enzymatic detoxification, fumonisin esterase FumD, enzyme kinetics, maize

## Abstract

Enzymatic detoxification has become a promising approach for control of mycotoxins postharvest in grains through modification of chemical structures determining their toxicity. In the present study fumonisin esterase FumD (EC 3.1.1.87) (FUM*zyme*^®^; BIOMIN, Tulln, Austria), hydrolysing fumonisin (FB) mycotoxins by de-esterification, was utilised to develop an enzymatic reduction method in a maize kernel enzyme incubation mixture. Efficacy of the FumD FB reduction method in “low” and “high” FB contaminated home-grown maize was compared by monitoring FB_1_ hydrolysis to the hydrolysed FB_1_ (HFB_1_) product utilising a validated LC-MS/MS analytical method. The method was further evaluated in terms of enzyme activity and treatment duration by assessing enzyme kinetic parameters and the relative distribution of HFB_1_ between maize kernels and the residual aqueous environment. FumD treatments resulted in significant reduction (≥80%) in “low” (≥1000 U/L, *p* < 0.05) and “high” (100 U/L, *p* < 0.05; ≥1000 U/L, *p* < 0.0001) FB contaminated maize after 1 h respectively, with an approximate 1:1 µmol conversion ratio of FB_1_ into the formation of HFB_1_. Enzyme kinetic parameters indicated that, depending on the activity of FumD utilised, a significantly (*p* < 0.05) higher FB_1_ conversion rate was noticed in “high” FB contaminated maize. The FumD FB reduction method in maize could find application in commercial maize-based practices as well as in communities utilising home-grown maize as a main dietary staple and known to be exposed above the tolerable daily intake levels.

## 1. Introduction

The lack of effective and environmentally safe chemical control methods against fungal infection and mycotoxin production in maize initiated investigations into biologically safe alternatives to prevent these contaminants from entering the food chain [1]. Methods involve the application of natural resources, including plant material, microbial cultures, genetic material, clay minerals and enzymes [2,3,4,5,6,7,8,9,10,11]. Enzymatic degradation of mycotoxins in food sources, postharvest, is a new research approach, providing many opportunities for novel initiatives to improve the safety of food with respect to mycotoxin reduction [1,12]. The focus is on targeted modification of the chemical structures by enzymatic cleavage or conversion of chemical bonds/groups that play a key role during cytotoxicity [13,14]. Detoxification of the fumonisins is achieved by enzymatic deamination of the free amino group at C-2 and de-esterification of the ester bonds at C-14 and C-15. A knowledge base on reduction of mycotoxin concentrations by bacterial and fungal cultures has been established over the years [14,15,16,17]. This information was further developed by identifying microbial enzymes responsible for detoxification, characterization of genes encoding the enzymes, expression of genes in food-grade microorganisms, and development of culture and recombinant enzyme preparations for commercial application. Microorganisms and enzymes capable of degrading fumonisin B_1_ (FB_1_) include carboxylesterase and amino oxidase enzymes of *Exophiala spinifera* ATCC 74269, *Rhinocladiella atrovirens* ATCC 74270 as well as carboxylesterase and aminotransferase enzymes of Bacterium ATCC 55552 and *Sphingopyxis macrogoltabida* MTA144 [14,15,16,17,18,19].

Recently, a commercial fumonisin esterase FumD (EC 3.1.1.87) (FUM*zyme*^®^; BIOMIN, Tulln, Austria) capable of effectively hydrolysing the tricarballylic acid groups of FB_1_ yielding hydrolysed FB_1_ (HFB_1_), was introduced. The enzyme activity is specific and irreversible, while HFB_1_ exhibited less toxic effects when evaluated in pig intestine, as indicated by disruption of the sphinganine/sphingosine ratios in the liver and plasma, induction of an intestinal immune response, the absence of hepatotoxicity, and changes in intestinal morphology [20]. FUM*zyme*^®^ has been regarded safe for humans, animals and the environment by the European Food Safety Authority (EFSA) [21]. Although enzymatic detoxification has become a promising approach and found application in the animal feed industry [21,22,23,24], a broader application to safeguard human food still has to be investigated. Such an approach could add value to commercial maize-based manufacturing processes during which the fumonisins are concentrated in certain products and co-products, as well as in subsistence farming communities where people are exposed to unacceptable levels of the fumonisins in their staple diet.

The present study describes the development of a FumD FB reduction method utilising home-grown maize batches containing “low” and “high” levels of FB. The efficacy of the reduction method was determined by monitoring the extent of FB_1_ hydrolysis and the formation of HFB_1_ in residual maize kernels and enzyme solutions utilising a validated LC-MS/MS analytical method. The FumD FB reduction method was optimized in terms of enzyme activity and treatment duration considering specific enzyme kinetic parameters to evaluate differences between maize samples with varying FB levels. Distribution of HFB_1_ between the kernel and aqueous phase was also recorded.

## 2. Results

### 2.1. Validation of The Extraction and Chromatographic Quantification Methods

The method validation parameters are summarised in Table 1 and Table 2. The lower limit of quantification (LOQ) for each fumonisin was determined from a signal to noise (s/n) ratio of at least five times the response compared to the blank response. Analyte responses were identifiable, discrete and reproducible. LOQ values for the individual fumonisins ranged between 2.8–3.5 µg/kg. Linearity (r^2^) of the fumonisin calibration curve was >0.998. Selectivity of the method was confirmed by demonstrating no interfering peaks at LOQ for each of the fumonisins. Accuracy was determined by replicate analysis of samples containing known amounts of analyte, resulting in means within 15% from the theoretical values. Recoveries were calculated by comparison of the response obtained for each mycotoxin with that of known spiked concentrations in blank maize, expressed as a percentage. The recoveries for the individual fumonisins ranged from 79–84% and were consistent and reproducible. Fumonisin concentrations in the FAPAS quality control reference maize samples were always within the stipulated ranges for each fumonisin (data not shown).

### 2.2. FB and HFB_1_ Concentrations in Maize

**Figure 1:**
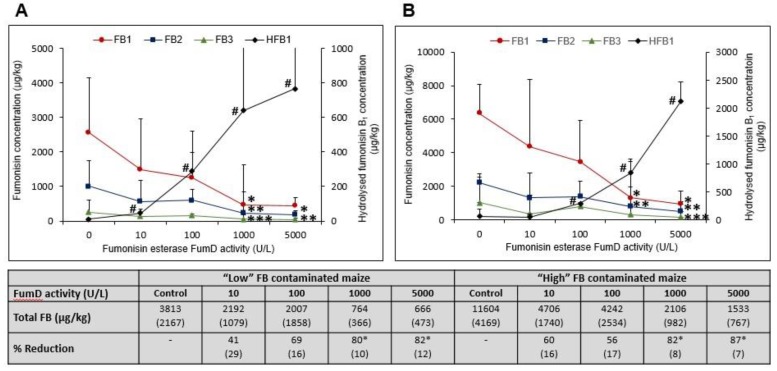
Fumonisin B (FB_1_, FB_2_, FB_3_) and hydrolysed fumonisin B_1_ (HFB_1_) concentrations (µg/kg) maize as a function of fumonisin esterase FumD activity (0, 10, 100, 1000 and 5000 U/L) after 1 h treatment. (**A**) “Low” and (**B**) “High” FB contaminated maize. Values represent means of three to five replications of experiments and error bars indicate standard deviations. The statistical analyses are based on natural log (ln) transformations. The *, **, *** and ^#^ indicate significant (*p* < 0.05) differences of means from the water control (0 U/L) treatments. Table insert: % total reduction of the total FB as a function of enzyme actvity after 1 h in “low” and “high” FB contaminated maize. Standard deviations are in brackets.

FB and HFB_1_ concentrations (µg/kg) (mean values and standard deviations in brackets) in untreated “low” FB contaminated maize: FB_1_, 3326 (1588); FB_2_, 1503 (749); FB_3_, 530 (376); and HFB_1_, 182 (106). Concentrations in “high” FB contaminated maize: FB_1_, 9343 (1756); FB_2_, 3559 (541); FB_3_, 1701 (1557); and HFB_1_, 342 (137). FB concentrations in “low” and “high” FB contaminated FumD treated maize are summarised in Figure 1 (Table insert). The HFB_1_ levels in the “low” and “high” FB contaminated FumD treated maize were 12 (8) and 65 (59), respectively. High variation in the FB levels existed between subsamples within each of the two maize batches, which is a common phenomenon for this type of analyses mainly due to the random distribution of infected kernels throughout a specific sample [25,26]. 

### 2.3. Optimal FumD activity and Conversion Ratios (Fixed Time Incubation)

#### 2.3.1. FB_1_ Hydrolysis and Formation of HFB_1_ in Residual Maize Kernels as a Function of Fumd Activity 

The pH of maize-enzyme solutions before and after 1 h enzyme treatments were 5.1 ± 0.10 and 4.8 ± 0.04 respectively for “low” FB contaminated maize. For the “high” FB contaminated maize it was 5.7 ± 0.12 and 5.4 ± 0.04. 

When compared to the water control sample in the absence of the enzyme, the total FB (FB_1_, FB_2_ and FB_3_) concentrations were reduced as a function of an increased enzyme activity during a 1 h incubation in both the “low” and “high” FB contaminated maize (Figure 1A,B). Treatment with ≥1000 U/L resulted in an 80% reduction in total FB concentrations (Figure 1, table insert) when compared to the water control. Incubations of “low” FB contaminated maize with 10 and 100 U/L FumD markedly (*p* > 0.05) reduced FB_1_ when compared to the water control incubation (Figure 1A). A significant reduction in FB_1_ was observed with 1000 U/L and 5000 U/L when compared to the water control (*p* < 0.05), with no significant difference (*p* > 0.05) between the two incubations. The reduction in FB_1_ concentrations coincided with a significant increase in HFB_1_ concentrations from 10 U/L (*p* < 0.01) to 1000 U/L (*p* < 0.001) and 5000 U/L (*p* < 0.001), when compared to the water control. Only trace amounts of HFB_1_ were detected in the water control samples of “low” FB contaminated maize.

In “high” FB contaminated maize, a significant reduction in FB_1_ was observed at FumD activities of 100 U/L (*p* < 0.05), 1000 U/L (*p* < 0.0001) and 5000 U/L (*p* < 0.0001) when compared to the water control with no significant (*p* > 0.05) differences between the different incubations (Figure 1B). The total FB concentrations (Figure 1B Table insert) were again reduced by ≥80% at the two highest FumD concentrations. Incubation with 10 U/L FumD only markedly reduced the FB_1_ concentration while incubation with 1000 U/L resulted in 79% reduction in FB_1,_ and 82% reduction in total FB concentrations, respectively. The reduction in FB_1_ again coincided with a significant (*p* < 0.0001) increase in HFB_1_ concentrations with treatments ≥100 U/L, while only trace amounts of HFB_1_ were detected in the water control. 

#### 2.3.2. FB_1_ Hydrolysis and Formation of Hfb_1_ in the Residual Solutions as a Function of Fumd Activity

**Figure 2:**
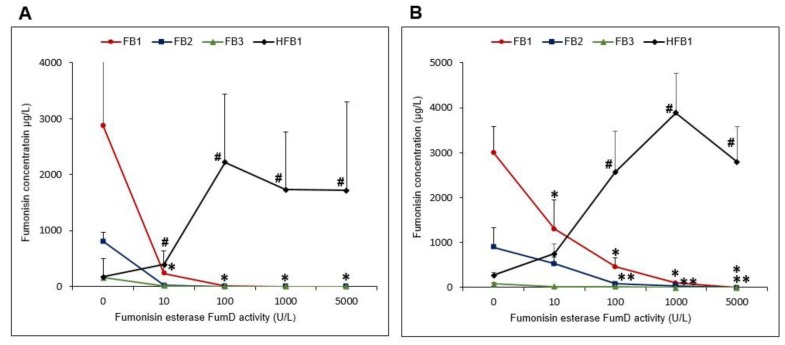
FB (FB_1_, FB_2_, FB_3_) and hydrolysed fumonisin B_1_ (HFB_1_) concentrations in residual water control and enzyme solutions as a function of fumonisin esterase FumD activity (0, 10, 100, 1000 and 5000 U/L) after 1 h treatment. (**A**) “Low” and (**B**) “High” FB contaminated maize. Values represent means of three to five replications of experiments and error bars indicate standard deviations. The statistical analyses are based on natural log (ln) transformations. The *, ** and ^#^ indicate significant (*p* < 0.05) differences of means from the respective water control (0 U/L) treatments.

Residual water control samples obtained from the “low” FB contaminated maize contained high FB and low HFB_1_ concentrations (Figure 2A). FumD reduced (*p* < 0.05) FB as a function of increased enzyme activity. The reduction in FB_1_ coincided with a significant increase in HFB_1_ from 10 U/L (*p* < 0.01) reaching a maximum at 100 U/L with no further increase >100 U/L. Treatment with 10 U/L resulted in an almost complete removal of FB_1_, with almost no FB_1_ detected ≥100 U/L. Complete reduction of FB_2_ and FB_3_ was also observed ≥10 U/L. A similar trend was observed in residual water control and enzyme solutions of the “high” FB contaminated maize, although FB_1_ was only completely removed >1000 U/L (Figure 2B). Total reduction of FB_2_ and FB_3_ was observed with ≥100 U/L FumD activity. A significant (*p* < 0.05) increase in HFB_1_ was noticed ≥10 U/L, reaching a maximum at 100 U/L.

#### 2.3.3. FB_1_ Hydrolysis Relative to the Formation of HFB_1_

When considering the total mean FB_1_ levels in the incubation mixtures (levels in residual maize kernels plus residual solutions expressed in micromoles), it was markedly to significantly (*p* < 0.05) higher in “high” FB contaminated maize although it varied when utilizing the different enzyme incubations (Table 3). Although the mean FB_1_ converted were similar between both samples treated with the various FumD activities, the % FB_1_ (µmol) loss was significantly (*p* < 0.05) higher in “low” FB contaminated maize at the two lower enzyme activities with no difference when using higher FumD activities (≥1000 U/L). Overall, FumD significantly (*p* < 0.05) increased the mean total HFB_1_ levels in both samples as a function of the enzyme activity reaching a maximum level ≥100 U/L. When considering the FB_1_:HFB_1_ conversion ratio, FumD resulted in a markedly lower conversion at 10 U/L in “low” as compared to “high” FB contaminated maize. The FB_1_:HFB_1_ conversion ratios were similar with 100 U/L, reaching approximately a 1:1 ratio at ≥1000 U/L, implying maximum conversion. 

#### 2.3.4. Relative HFB_1_ Distribution in Residual Maize Kernels and Solutions Following FumD Incubations

The total HFB_1_ in the incubation mixture increased significantly (*p* < 0.05) in incubation ≥100 U/L FumD activities with no difference at higher enzyme activities and/or between the “low” and “high” FB contaminated maize samples. The % HFB_1_ accumulation in the “low” FB contaminated maize increased as a function of increased FumD activity reaching a maximum level at the 1000 U/L (Table 4). A similar response was noticed in the “high” FB contaminated maize showing a typical dose response effect with a significant (*p* < 0.05) higher level obtained with 5000 U/L FumD activity. Maximum levels when considering the % total mean HFB_1_ were noticed in the residual solution up to 100 U/L FumD, which markedly and significantly decreased in the “low” and “high” FB contaminated samples at higher FumD activities, respectively. 

### 2.4. Comparative Enzyme Kinetics of FB_1_ Conversion as a Function of Time and FumD Activity 

#### 2.4.1. FB_1_ Leaching into the Aqueous Phase (Water Control Treatment)

**Figure 3:**
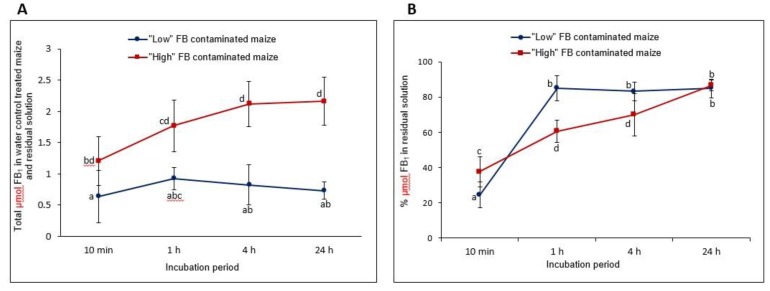
Total amount of FB_1_ leaching into the incubation mixture in the absence of the enzyme as a function of time, of “low” and “high” FB contaminated maize (**A**). The % µmol FB_1_ leaching into the residual water during a comparable time of incubation (**B**). Values represent means ± standard deviations of three to five replications of experiments. Statistical analyses are based on natural log (ln) and differences (*p* < 0.05) between treatment periods within and between “low” and “high” FB contaminated maize are indicated with different letters.

The total mean baseline FB_1_ levels, i.e., total FB_1_ (µmol) in the “low” and “high” FB contaminated maize incubation mixtures as a function of the different incubation times (10 min, 1 h, 4 h and 24 h) are summarised in Figure 3. A significantly (*p* < 0.05) higher total FB_1_ was noticed in the total incubation mixture utilising the “high” FB contaminated maize at each time point (Figure 3A). FB_1_ leaching from “low” FB contaminated maize, reached saturation after 1 h as, despite that different samples were used, no further increase was noticed in the samples incubated for 4 and 24 h (Figure 3B). In contrast, leaching of FB_1_ from the “high” FB contaminated maize, although significantly (*p* < 0.05) lower after 1 h and 4 h, steadily increased until it mimicked the level of the “low” FB contaminated maize after 24 h.

#### 2.4.2. Comparative Enzyme Kinetics of FB_1_ Hydrolysis 

The conversion rates of FB_1_ into HFB_1_, expressed as nmol/min/mg enzyme, following the 100 and 1000 U/L FumD incubations as a function of time are summarised in Table 5. The FB_1_ hydrolysis rates in the presence of 100 U/L and 1000 U/L FumD was maximum after 10 min in both maize samples and was significantly higher (*p* < 0.05) in the “high” FB contaminated maize. The hydrolysis rates decreased from 10 min to 24 h in the maize samples for both enzyme activities. The HFB_1_ formation rate followed the same trend. Overall, the FB_1_ hydrolyses and HFB_1_ formation rates were significantly (*p* < 0.05) decreased in the 1000 U/L as compared to 100 U/L FumD incubations.

#### 2.4.3. Comparative of FB_1_ Hydrolysis to HFB_1_ Formation Ratios

There was a significant (*p* < 0.05) difference between the conversion rates (FB_1_ hydrolysis (nmol/min/mg enzyme)) of the 100 and 1000 U/L FumD incubations during each respective treatment period in both “low” and “high” FB contaminated maize. In both maize batches the 10 min incubation with 100 U/L exhibited a significant (*p* < 0.05) higher conversion rate when compared with the longer incubations (Table 5). The conversion ratio (FB_1_ hydrolysis: HFB_1_ formation) also tended to be increased in the “low” compared to the “high” FB contaminated maize with the 100 U/L incubation. A similar trend in the conversion ratios was obtained during the 1 h enzyme treatment study at the lower FumD enzyme activities (Table 3). 

## 3. Discussion

Bioremediation, utilising enzymes for detoxification and/or degradation of environmental chemicals has become an intuitive way to reduce the risk of exposure in animals and humans. A popular approach is to use enzymes that can bind with high affinity toxic compounds and catalyze their hydrolysis, thereby abandoning or reducing their toxicity [27]. The present study established an enzymatic method for FB reduction utilizing fumonisin esterase FumD in whole maize intended for human consumption. The method involved treatment of “low” and “high” FB contaminated home-grown maize with varying FumD activities in solution as a function of time. FumD has been proved effective as a feed enzyme when incorporated into ground maize at a concentration of 40 U/kg feed [23]. In the current method, FumD was applied to whole maize, therefore the accessibility of FB would be expected to require higher FumD activities and longer incubation times for reduction than with ground maize. However, a major advantage of the current FumD FB reduction method is that the bulk of the residual enzyme and hydrolysed less toxic HFB_1_ product are not associated with the treated maize kernels, which could find application when considering the reduction of FBs in food intended for human consumption. 

Despite the fact FumD treatments were performed in the current reduction method at sub-optimal pH (approximately pH 5 to 5.5) and ambient temperature (±23 °C) conditions [optimal enzyme activity is obtained at pH 8 and 30 °C [14], effective hydrolysis of FB was observed in both maize batches. Treatment with 1000 U/L FumD effectively hydrolysed FB in “low” and “high” FB contaminated maize resulting in ≥80% reduction in the total FB levels after 1 h while lower enzyme activities (≤100 U/L) were less effective. At lower enzyme activities large variations in the FB levels and hydrolyses rates were noticed, which could be related to (i) sample variation within maize batches, (ii) enzyme and/or substrate availability as a function of time, and (iii) the formation of partially hydrolysed FB (PHFB) [28], which will be present as transient intermediates to the completely hydrolysed molecule at high enzyme activities. A significant (*p* < 0.05) decrease of FB_1_ reduction in the “low” FB contaminated maize was noticed already at 10 U/L FumD compared to the 100 U/L in the “high” FB contaminated maize. These differences could be related to the FumD activities applied, FB_1_ availability at the kernel/aqueous interphase and the formation of PHFB, and will be discussed below.

When considering differences in the formation of HFB_1_ into the residual enzyme solution, maximum levels were already noticed from 100 U/L FumD activity. This suggests that some of the hydrolysed product is retained within the maize matrix as well as the possible formation of PHFB_1_. This became apparent as, although HFB_1_ was mainly associated (>90%) with the residual enzyme solution at low enzyme activities (10 and 100 U/L), it tends to accumulate in the “high” and to a lesser extent in the “low” FB contaminated maize kernels at incubations using increased FumD activities (≥1000 U/L). This could be ascribed to the formation of HFB_1_ and PHFB_1_ in the inner layers of the maize kernel matrix as compared to the “low” FB contaminated maize, where it mainly occurred on the surface. 

As mentioned above, differences occurred in the extent of FB_1_ hydrolysis between the two samples when considering the % FB_1_ loss in the maize as well as the FB_1_ hydrolysis to HFB_1_ formation ratio in the incubation mixture, specifically at the lower FumD enzyme activities. These differences were also noticed when considering the enzyme kinetics of FB_1_ hydrolysis and HFB_1_ formation rates at 100 and 1000 U/L FumD activities, which were significantly (*p* < 0.05) increased in the “high” FB contaminated maize. The higher FB_1_ conversion rate obtained with the 100 U/L FumD activity as compared to the 1000 U/L enzyme activity is related, as mentioned above, to FB availability and/or the limitation thereof when utilising excess enzyme activities.

The level of FB contamination determined the hydrolysis rate implying that fungal infiltration and FB production inside the kernel are key rate limiting factors. This became evident as *Fusarium* spp. infects maize with the fumonisins concentrated in the pericarp and embryo of the maize kernel [29], while in damaged kernels the fungus is likely to penetrate deeper into the kernel, contaminating the endosperm [30,31,32]. It is known that leaching of substances from the maize endosperm occurs during absorption of water while damage to the kernel pericarp increases leaching early during water absorption [33].

Differences in FB_1_ hydrolysis rates between the “low” and “high” FB contaminated maize batches are therefore likely to depend on the leaching of FB_1_ from the maize kernel and the interaction of FumD at the kernel outer layer/aqueous inter phase. This became apparent when considering the % FB_1_ hydrolysis in the “low” FB contaminated sample that reached a maximum already after 100 U/L compared to the “high” FB contaminated maize reaching a maximum at 1000 U/L. This implies that the FB_1_ became depleted as a substrate for the enzyme much earlier. This was also evident as leaching of FB_1_ into the water control was faster from the “low” FB contaminated maize at 1 and 4 h of incubation compared to a more gradual effect considering the “high” FB contaminated maize. The gradual increased leaching of FB_1_ from the “high” FB contaminated maize could be related to the extent of fungal damage of the kernels that will determine the diffusion rate of FB_1_ from the inner layers. Therefore, the higher initial (10 min) conversion rate of FB_1_ in the “high” as compared to “low” FB contaminated maize is facilitated by the accessibility of FB_1_ associated with the inner as well as outer layers. The location of FB_1_ in the kernel and the leaching rate into the aqueous enzyme-solution could therefore affect the efficacy of FB conversion rates and substrate to product ratios as noticed in the current study. However, the efficacy of FumD is not only determined by the concentration of FB_1_ leaching into the aqueous phase but could also depend on the enzyme penetration into the endosperm which could explain the differences in FB conversion rates between “low” and “high” FB contaminated maize. This became evident as an increased % of HFB_1_ was noticed in the maize kernels when utilising higher FumD activities. Therefore, depending on the extent of fungal damage and FB contamination to the maize pericarp/endosperm, a specific FumD activity seems to be required for maximum FB_1_ hydrolysis, yielding a 1:1 FB_1_ hydrolysis to HFB_1_ formation ratio. This would become important when considering the application of the enzyme in different technological approaches utilising maize samples with varying FB contamination levels.

From a technological perspective, enzymatic detoxification of FB has been utilised effectively in the animal feed industry [21,22,24], while very little is known about possible applications to reduce exposure in humans. In this regard, commercial maize-based manufacturing processes, including dry milling and ethanol production are challenged with maintaining regulatory levels for FB in products and co-products [29,34]. During dry milling the FB mycotoxins are known to be ± 3-fold concentrated in the surface layers and are mainly confined to the total hominy feed fractions, which are mainly used in animal feed or non-food products [29]. As most micronutrients are concentrated in total hominy feed and a large portion of maize grain is lost in this fraction [35], FB decontamination could be of value with respect to human food. It could also find application during ethanol production, as the co-products wet and dry distillers’ grains and solubles, containing high levels of FB, are increasingly being marketed as protein-rich and cost-saving inclusion in livestock and poultry feed [34]. Therefore, possible economic advantages of the FumD FB reduction method for commercial maize-based manufacturing processes should be further investigated.

The FumD FB reduction method in maize could also find application in communities utilising home-grown maize as a main dietary staple and known to be exposed above the tolerable daily intake levels, i.e., a PMTDI of 2 µg/kg/bw/day [36,37]. To decrease the risk of FB exposure, culturally sensitive, practical and biologically based methods of reduction are relevant and need to be implemented [1]. In the current study, the “low” FB contaminated maize contained FB levels below the regulatory maximum levels for fumonisins in maize (total FB_1_ and FB_2_ 4000 µg/kg) set by the Codex Alimentarius Commission [38], while the high FB contaminated samples exceeded that. These maize batches were therefore ideal to evaluate the newly designed FumD FB reduction method, which could find application in maize subsistence farming communities where exposure to high levels of FB is the norm on a daily basis [37,39]. Of interest is that the bulk of HFB_1_ resides into the aqueous phase, which will further minimize exposure to, not only FB_1_, but also to the less toxic breakdown product, HFB_1_. However, increased amounts (up to 32%) of HFB_1_ accumulated in “high” FB contaminated maize in the presence of increased FumD activities.

Recently a practical and culturally sensitive maize hand-sorting and water wash intervention method resulted in 84% reduction of FB_1_ levels [40]. In the current study, the 1000 U/L FumD treatment resulted in access of 80% reduction in total FBs in both “low” and “high” FB contaminated samples. However, as only approximately 10% of FB is removed from the maize kernels during the water wash procedure [41], the newly developed FumD FB reduction method could effectively be applied to further reduce FB exposure prior to food preparation. 

## 4. Conclusions

The present study developed an innovative FumD FB reduction method in whole maize. The method is suitable for direct application in the food chain postharvest, resulting in the reduction of FB_1_ with the formation of the hydrolysed breakdown product, HFB_1,_ mainly associated with the aqueous phase to be discarded. Reduction of FB in contaminated maize could have a positive impact on food safety and security as well as having economic benefits during manufacturing processes.

## 5. Materials and Methods

### 5.1. Chemicals

Methanol, acetonitrile, formic acid (HPLC grade) and Whatman filter paper were obtained from Merck (Kenilworth, NJ, USA). Water for all experiments was successively purified by reverse osmosis followed by Milli-Q water purification (Millipore, Burlington, MA, USA).

### 5.2. Fumonisin Standard Solutions

Pure analytical standards of FB_1_, FB_2,_ FB_3_ and HFB_1_ (purity > 97%) were prepared at the Institute of Biomedical and Microbial Biotechnology of the Cape Peninsula University of Technology, South Africa, according to the methods of Cawood et al. [42] and Gelderblom et al. [13]. Stock solutions of the individual purified fumonisin standards were prepared (1 mg/mL in acetonitrile-H_2_O (1:1)) and aliquots used to prepare an evaporated working solution containing the fumonisin standards at individual concentrations of 5 µg/mL. For compiling matrix-matched calibration curves, five working standard dilutions were prepared with blank maize matrix extract as solvent, as described below.

### 5.3. Maize Sample Collection

Home-grown maize was collected in the Centane, Mnquma Local Municipality (areas of the former Transkei region) of the Eastern Cape Province, South Africa from households of subsistence maize farmers. “Good” home-grown whole maize was collected directly from visibly healthy batches destined for human consumption. “Mouldy” home-grown whole maize was collected directly from “mouldy” batches destined for chicken/livestock feed. The “good” and “mouldy” maize batches were labelled “low” and “high” FB contamination, respectively. Each maize batch was thoroughly mixed and kept at 4 °C until analysed. Control maize containing no fumonisins, was obtained from the Southern African Grain Laboratory (Pretoria, South Africa) and used for the preparation of a maize extract used for matrix-matched calibration curves. 

### 5.4. FumD Enzyme Preparation

A fumonisin esterase, designated FumD (EC 3.1.1.87; FUM*zyme*^®^), was obtained from BIOMIN (Tulln, Austria) with a specific activity of 13,400 U/g. One unit is the enzymatic activity defined to release 1 µmol tricarballylic acid per minute from 100 µM FB_1_ in 20 mM Tris-HCl buffer pH 8.0 containing 0.1 mg/mL bovine serum albumin at 30 °C. A stock enzyme solution (400 U/mL) was prepared in the Tris-HCl buffer and used in all experiments. 

### 5.5. The FumD FB Reduction Method 

Enzyme solutions utilised were prepared in distilled water from the FumD stock solution. Maize kernels (100 g) of the “low” and “high” FB contaminated maize batches were weighed in Erlenmeyer flasks (500 mL) and enzyme solution added (200 mL), obtaining a maize to solvent ratio of 1:2. Samples were mixed at 80 rpm on a shaker (New Brunswick Scientific, Edison Township, NJ, USA) at ambient temperature (± 23 °C) for different incubation periods. The residual enzyme solution was decanted and stored at −20 °C until analysed, while the residual maize kernels were dried onto laboratory paper towels at ambient temperature for 48 h. Residual maize kernel samples were ground in a laboratory mill (Falling Number AB, Stockholm, Sweden) to a fine meal and kept in airtight containers at −20 °C until analysed. Three to five replicates were included for each treatment. 

### 5.6. FumD Incubation Protocols

To obtain the optimal enzyme activity required for FB hydrolysis the maize samples were incubated with different FumD activities (10, 100, 1000 and 5000 U/L) for 1 h as described above. The pH values of the maize-enzyme solutions were determined before and after the incubation treatment. In a follow-up experiment two FumD activities (100 and 1000 U/L) were used, and incubations were carried out over varying time periods including 10 min, 1 h, 4 h and 24 h to investigate the FB hydrolysis kinetics. Reference water control incubations were included in each experiment. All samples were processed and stored as described above.

### 5.7. Analyses of FB and HFB_1_ Concentrations in Maize and Residual Solutions

FB_1_, FB_2,_ FB_3_ and HFB_1_ concentrations were determined in the (i) untreated “low” and “high” FB contaminated maize batches (five replicates), (ii) the water control and enzyme treated residual maize kernel samples, and (iii) residual water control and enzyme solutions (3–5 replicate experiments). 

#### 5.7.1. Extraction Methods

FB and HFB_1_ were extracted from maize according to the method of Sewram et al. [43] with minor modifications. Briefly, 100 mL of extraction solvent [methanol: acetonitrile: water (25:25:50; *v*/*v*/*v*)] was added to ground maize kernels (10 g) and placed on a shaker (80 rpm) for 20 min. The extracts were subsequently centrifuged (4000× *g*) in a refrigerated Sorvall RC-3B centrifuge (DuPont, Norwalk, CT, USA) at 4 °C for 10 min. The supernatant (20 ml) was diluted (1:1) with methanol:water (25:75), filtered (Whatman No 4 filter paper) and filtrates analyzed by direct injection into the LC-MS/MS. FAPAS (London, UK) quality control reference maize samples (Cat no T22123QC), containing the mycotoxins in known concentration ranges were included. For analyses of the residual solutions, samples were filtered (Whatman No 4 filter paper) and filtrates analysed directly by LC-MS/MS. Matrix-matched standard solutions for calibration curves were prepared utilising an extract prepared from control maize.

#### 5.7.2. Chromatographic Quantification of FB and HFB_1_

Quantification of FB and HFB_1_ in maize extracts and residual enzyme solutions was performed by the Mass Spectrometry Unit of the Central Analytical Facility of Stellenbosch University, South Africa. The mycotoxins were separated on a reversed-phase BEH C_18_ column (2.1 × 100 mm; particle size 1.7 µm) (Waters, Milford, MA, USA) and analysed with positive electrospray ionisation (EI) in the multiple reaction monitoring (MRM) mode in a Waters Acquity Ultra high performance Liquid Chromatograph (UPLC) coupled to a Tandem Quadrupole Mass spectrometer (Waters Xevo TQ MS). Eluent A was water and eluent B was methanol, both containing 0.1% formic acid. The chromatographic method held the initial mobile phase composition (15% B) constant for 2 min, followed by a linear gradient to 100% B within 3 min. This final condition was held for 3.30 min, followed by 8 min of column re-equilibration at 15% B. The flow rate of the mobile phase was 0.35 ml/min. For each compound, one precursor and two product ions were monitored, one product ion for quantification and one for confirmation. A calibration curve consisting of five matrix-matched standards for each mycotoxin was used for quantification.

#### 5.7.3. Validation of the Extraction and FB Quantification Methods

The extraction and chromatographic methods were validated by determining the LOQ, linearity (r^2^) of the calibration curve, selectivity, accuracy, and % recovery according to guidelines of the United States Department of Health and Human Services, Food and Drug Administration [44]. 

### 5.8. Statistical Analyses

The NCSS Version 11 software [45] was used for statistical analysis. Data were subjected to natural log (ln) transformation of all variables and analysed within a generalised linear model ANOVA. Multiple comparisons were analysed using the Tukey-Kramer’s multiple comparison procedure. This method provides joint simultaneous confidence intervals for all pairwise differences between the means; and also provides the multiple comparison *p*-value. Generally, *p* < 0.05 was used as statistical significance. In addition, the size of the F-ratios was used to measure relative sizes of differences.

## Figures and Tables

**Table 1:** LC-MS/MS conditions for quantification of fumonisins and hydrolysed fumonisin b_1_ by positive ESI at 3.5 kv capillary voltage.

Analyte	Cone Voltage	Precursor	Quantifier (Collision Energy)	Qualifier (Collision Energy)
Fumonisin B_1_	50	722.3	334.3 (40)	352.3 (38)
Fumonisins B_2_ and B_3_	50	706.3	318.3 (40)	336.3 (40)
Hydrolysed fumonisin B_1_	25	406.6	334.3 (25)	352.4 (20)

**Table 2:** Validation of the analytical method for fumonisin analyses in maize.

Analyte	LOQ (µg/kg)	Spike Level (µg/kg)	Recovery (%)	RSDr (%)
Fumonisin B_1_	3.5	1060	84	2
Fumonisin B_2_	2.8	925	66	4
Fumonisin B_3_	2.8	520	79	1
Hydrolysed fumonisin B_1_	2.8	800	80	2

LOQ, lower limit of quantification; RSDr, relative standard deviation for repeatability.

**Table 3:** Comparative FB_1_ conversion relative to HFB_1_ formation between “low” and “high” FB contaminated maize during 1 h incubation with different FumD activities.

FumD Aactivity (U/L)	Total Mean µmol FB_1_ in Incubation Mixture *	Mean µmol FB_1_ Converted	Mean µmol FB_1_ Loss (%)	Total Mean µmol HFB_1_ in Incubation Mixture	FB_1_:HFB_1_ µmol Conversion Ratio
**“Low” FB Contaminated Maize**					
0 **	1.15 ± 0.55	-	-	0.02 ± 0.01	42.75 ± 12.14
10	0.27 ± 0.14 ^ab^	0.88 ± 0.14 ^a^	76.58 ± 11.90 ^abc^	0.13± 0.05 ^a^	7.22 ± 2.40 ^a^
100	0.18 ± 0.16 ^ac^	0.98 ± 0.16 ^a^	84.73 ± 14.14 ^ab^	0.94 ± 0.62 ^b^	2.52 ± 0.84 ^ab^
1000	0.07 ± 0.03 ^c^	1.09 ± 0.03 ^ab^	94.32 ± 2.91 ^a^	0.99 ± 0.60 ^b^	1.30 ± 0.58 ^bc^
5000	0.06 ± 0.05 ^c^	1.09 ± 0.05 ^ab^	94.64 ± 3.98 ^ab^	1.02 ± 1.00 ^b^	0.66 ± 0.32 ^c^
**“High” FB Contaminated Maize**					
0 **	1.74 ± 1.32	-	-	0.16 ± 0.10	10.23 ± 3.96
10	0.97 ± 0.50 ^b^	0.76 ± 0.50 ^a^	55.04 ± 17.68 ^c^	0.24 ± 0.15 ^a^	3.4 ± 1.73 ^abd^
100	0.71 ± 0.37 ^b^	1.02 ± 0.37 ^a^	58.84 ± 21.59 ^c^	1.17 ± 0.53 ^b^	1.38 ± 0.72 ^bc^
1000	0.21 ± 0.16 ^ac^	1.52 ± 0.16 ^b^	87.75 ± 9.31 ^ab^	1.96 ± 0.89 ^b^	0.93 ± 0.46 ^c^
5000	0.13 ± 0.07 ^ac^	1.61 ± 0.07 ^b^	92.61 ± 4.09 ^ab^	1.68 ± 0.66 ^b^	1.11 ± 0.54 ^cd^

The statistical analyses are based on natural log (ln) transformations. Values represent means ± standard deviations of three to five replications of experiments. Statistical differences (*p* < 0.05) in a column for FumD activities within and between the “low” and “high” FB contaminated maize are indicated with different letters. * Combined FB_1_ levels in maize and residual enzyme solution. ** Shaded areas represent the water control treatment (0 U/L). HFB_1_ levels were corrected accordingly in the presence of the enzyme.

**Table 4:** Distribution of HFB_1_ between residual maize and the solution following FumD treatment of “low” and “high” FB contaminated maize for 1 h.

FumD Activity (U/L)	Total Mean HFB_1_ (µmol) in 100 g Residual Maize Kernels	Total Mean HFB_1_ (µmol) in 200 mL Residual Solution	Mean µmol HFB_1_ in Residual Maize Kernels (%)	Mean µmol HFB_1_ in Residual Solution (%)
	**“Low” FB Contaminated Maize**			
10	0.01 ± 0.01 ^a^	0.13 ± 0.04 ^a^	5.65 ± 3.47 ^a^	94.35 ± 3.47 ^a^
100	0.07 ± 0.06 ^b^	0.87 ± 0.58 ^b^	7.35 ± 3.28 ^a^	92.65 ± 3.28 ^a^
1000	0.15 ± 0.11 ^b^	0.83 ± 0.50 ^b^	15.82 ± 4.48 ^b^	84.18 ± 4.48 ^b^
5000	0.19 ± 0.22 ^b^	0.83 ± 0.78 ^b^	16.25 ± 3.86 ^b^	83.75 ± 3.86 ^b^
	**“High” FB Contaminated Maize**			
10	0.00 ^a^	0.24 ± 0.14 ^a^	0.48 ± 0.58 ^a^	99.52 ± 0.60 ^a^
100	0.08 ± 0.06 ^b^	1.09 ± 0.48 ^b^	6.75 ± 2.24 ^b^	93.25 ± 2.24 ^b^
1000	0.19 ± 0.09 ^bc^	1.77 ± 0.86 ^b^	10.68 ± 4.33 ^b^	89.32 ± 4.33 ^b^
5000	0.50 ± 0.17 ^c^	1.17 ± 0.65 ^b^	32.90 ± 11.85 ^c^	67.10 ± 11.90 ^c^

The statistical analyses are based on natural log (ln) transformations. Values represent means ± standard deviations of three to five replications of experiments. Statistical differences (*p* < 0.05) in a column of the % HFB_1_ distribution within “low” and “high” FB contaminated maize are indicated with different letters.

**Table 5:** Enzyme kinetic parameters regarding the conversion of FB_1_ to HFB_1_ by different fumonisin esterase FumD activities (U/L) as a function of treatment period (10 min, 1 h, 4 h and 24 h) in “low” and “high” FB contaminated maize.

	“Low” FB Contaminated Maize								“High” FB Contaminated Maize							
**Treatment Duration**	**10 min**	**1 h**	**4 h**	**24 h**	**10 min**	**1 h**	**4 h**	**24 h**	**10 min**	**1 h**	**4 h**	**24 h**	**10 min**	**1 h**	**4 h**	**24 h**
**FumD Activity**	**FB**_**1**_ **Hydrolysis** **(nmol/min/mg Enzyme)**				**HFB**_**1**_ Formation * **(nmol/min/mg Enzyme)**				**FB**_**1**_ **Hydrolysis** **(nmol/min/mg Enzyme)**				**HFB**_**1**_ Formation * **(nmol/min/mg Enzyme)**			
**100 U/L**	38.34 (1.54) ^a^	8.23 (0.96) ^b^	1.89 (0.04) ^c^	0.32 (0.01) ^d^	20.26 (10.75) _A_	5.15 (0.80) ^B^	2.12 (0.56) ^C^	0.32 (0.12) ^D^	59.19 (12.08) ^i^	13.18 (3.24) ^j^	4.42 (1.03) ^e^	0.92 (0.04) ^f^	21.10 (8.56) ^A^	14.55 (1.46) ^A^	4.86 (0.82) ^B^	1.18 (0.16) ^C^
**1000 U/L**	3.06 (0.62)^e^	0.89 (0.11) ^f^	0.20 (0.01) ^g^	0.03 (0.00) ^h^	2.78 (1.55) ^C^	0.87 (0.16) ^E^	0.25 (0.12) ^D^	0.04 (0.01) ^F^	4.53 (2.26) e	1.70 (0.19) ^k^	0.52 (0.05) ^l^	0.10 (0.00) ^m^	3.64 (2.02) ^B^	1.98 (0.46) ^C^	0.67 (0.21) ^E^	0.11 (0.02) ^D^
**FB**_**1**_ **Hydrolysis: HFB**_**1**_ **Formation Ratio**									**FB**_**1**_ **Hydrolysis: HFB**_**1**_ **Formation Ratio**							
**100 U/L**	3.46 (1.93) ^p^	1.39 (0.62) ^pq^	0.95 (0.30) ^qr^	0.93 (0.36) ^pr^	-	-	-	-	1.94 (0.16) ^pr^	0.91 (0.20) ^pq^	0.91 (0.14) ^qr^	0.95 (0.33) ^qr^	-	-	-	-
**1000 U/L**	1.45 (0.94) ^pqs^	1.04 (0.16) ^pq^	0.93 (0.38) ^qr^	0.92 (0.35) ^qr^	-	-	-	-	1.10 (0.29) ^pqs^	0.91 (0.26) ^qrs^	0.84 (0.26) ^qrs^	0.92 (0.24) ^qrs^	-	-	-	-

The statistical analyses are based on natural log (ln) transformations. Values represent means of three to five replications of experiments with standard deviations indicated in brackets. Statistical differences (*p* < 0.05) of FB_1_ hydrolysis rates and conversion ratios between FumD treatments (columns) as a function of time (rows) and for “low” and “high” FB contaminated maize are indicated with different lower case letters. Statistical differences (*p* < 0.05) of HFB_1_ formation rates between FumD treatments (columns) as a function of time (rows) and for “low” and “high” FB contaminated maize are indicated with different uppercase letters. *Corrected for HFB_1_ concentrations obtained in control (0 U/L) treatments in “low” and “high” FB contaminated maize and residual enzyme solutions. 100 U/L and 1000 U/L FumD represent 7.5 and 75 mg/L specific activity, respectively.
